# Downregulation of Focal Adhesion Kinase (FAK) by cord blood stem cells inhibits angiogenesis in glioblastoma

**DOI:** 10.18632/aging.100217

**Published:** 2010-11-07

**Authors:** Venkata Ramesh Dasari, Kiranpreet Kaur, Kiran Kumar Velpula, Dzung H. Dinh, Andrew J. Tsung, Sanjeeva Mohanam, Jasti S. Rao

**Affiliations:** ^1^ Departments of Cancer Biology and Pharmacology, University of Illinois College of Medicine at Peoria, Peoria, IL 61605, USA; ^2^ Departments of Neurosurgery, University of Illinois College of Medicine at Peoria, Peoria, IL 61605, USA

**Keywords:** angiogenesis, cord blood stem cells, glioblastoma, FAK, integrins

## Abstract

Angiogenesis involves the formation of new blood vessels by rerouting or remodeling existing ones and is believed to be the primary method of vessel formation in gliomas. To study the mechanisms by which angiogenesis of glioma cells can be inhibited by human umbilical cord blood stem cells (hUCBSC), we studied two glioma cell lines (SNB19, U251) and a glioma xenograft cell line (5310) alone and in co-culture with hUCBSC. Conditioned media from co-cultures of glioma cells with hUCBSC showed reduced angiogenesis as evaluated by *in vitro* angiogenesis assay using HMEC cells. Reduction in angiogenesis was associated with downregulation of FAK and integrin α_v_β_3_ in the co-cultures of glioma cells. Downregulation of FAK gene is correlated with downregulation of many angiogenesis-related genes, including Ang1, VEGFA and Akt. Under *in vivo* conditions, neovascularization by glioma cells was inhibited by hUCBSC. Further, intracranial tumor growth was inhibited by hUCBSC in athymic nude mice. Similar to *in vitro* results, we observed downregulation of FAK, VEGF and Akt molecules to inhibit angiogenesis in the hUCBSC-treated nude mice brains. Taken together, our results suggest that hUCBSC have the potential to inhibit the angiogenesis of glioma cells both *in vitro* and *in vivo*.

## INTRODUCTION

Angiogenesis is a key event in the progression of malignant gliomas [1,2]. It is a highly regulated process that involves a complex cascade of events. Because of the physiological and pathological importance of angiogenesis in glioblastoma, extensive research has been carried out to identify the factors that regulate this process. The expression of angiogenic factors by tumor cells promotes the establishment of a tumor vasculature that facilitates tumor growth. Vascular endothelial growth factor (VEGF) is the most potent growth factor implicated in the signaling pathway of glioma angiogenesis in that it promotes proliferation and migration of endothelial cells [3]. Focal adhesion kinase (FAK) is a cytoplasmic tyrosine kinase, which is involved in early integrin signaling and has been shown to regulate cell migration, proliferation, survival, and invasion in some cell types. Integrin recognition of an extracellular matrix ligand typically results in the clustering of the integrin in the cell membrane and, in a temporally related manner, the autophosphorylation of FAK on tyrosine 397 (activation), followed by the formation of focal adhesions at the submembranous region of the cell [4,5]. The ability of FAK to promote cell migration, proliferation, and invasion suggested that FAK could be necessary for endothelial cell sprouting and tube formation [6]. FAK influences the dynamic regulation of integrin-associated adhesions and the actin cytoskeleton that is tethered there through diverse molecular interactions. This, in turn, regulates cell migration by controlling the focal-complex assembly/disassembly cycle at the leading lamellipodia of migrating cells while also controlling adhesion disassembly at the trailing edge. As these processes are crucial components of cell migration, and therefore also of invasion by cancer cells, FAK might well be involved in the spread of cancer cells [7]. Also, FAK has been found to be expressed in angiogenic blood vessels of malignant astrocytomas where it might contribute to angiogenesis by enabling haptotactic migration towards ECM proteins [6].

However, much more work needs to be done to fully determine the role of FAK in tumorigenesis, which might vary in different tumor types. The causal role of FAK in tumor development combined with reports that increased FAK expression is associated with poor clinical outcome [8,9] indicates that FAK might be a useful therapeutic target [7,10]. In the present study, we studied the effect of human umbilical cord blood-derived mesenchymal stem cells (hUCBSC) on the role of FAK and its related moleculesin glioma cells *in vitro* and *in vivo*. We observed that hUCBSC downregulate FAK and its related moleculesin both *in vitro* and *in vivo* conditions, thereby disrupting the process of angiogenesis in glioma tumors.

## RESULTS

### Inhibition of angiogenesis by conditioned media of hUCBSC

**Figure 1.**
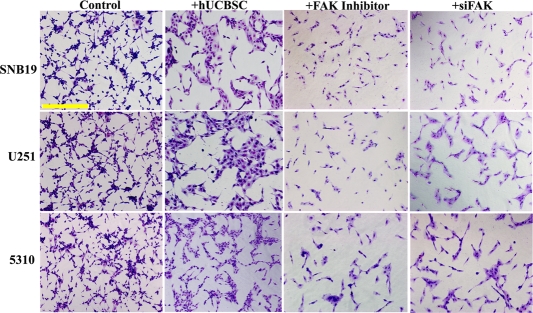
Inhibition of vessel formation by hUCBSC treatment. Conditioned medium was collected from the single and co-cultures (with hUCBSC) of glioma cells (SNB19, U251 and 5310) and added to HMEC cells pre-seeded (5000 cells/well) in 96-well plates. After 72h of incubation, HMEC cells were observed for capillary-like network formation and photographed under a light microscope. Bar = 500μm. Similarly, conditioned medium was collected from the glioma cells treated either with FAK inhibitor (10μM for 1.5h) or transfected with siFAK. Conditioned medium was added to HMEC cells pre-seeded (5000 cells/well) in 96-well plates. After 72h of incubation, HMEC cells were observed for capillary-like network formation and photographed under a light microscope.

**Figure 2.**
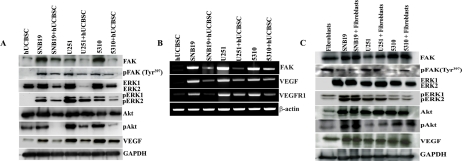
Downregulation of angiogenesis related proteins by hUCBSC. (**A**) Equal amounts of proteins (40μg) from the single and co-cultures were loaded onto 8-14% gels and transferred onto nitrocellulose membranes, which were then probed with respective antibodies. GAPDH was used a positive loading control. (**B**) Reverse-transcription based PCR analysis of single and co-cultures of glioma cells showing expression of FAK, VEGF, VEGFR1 and β-actin. (**C**) Single and co-cultures of glioma cells with fibroblasts showing expression of proteins by immunoblot analyses. The results are from 3 separate experiments.

To evaluate the effect of hUCBSC on glioma angiogenesis, we co-cultured glioma cells with hUCBSC for 72h. The conditioned medium from both single and co-cultures were collected and HMEC cells were grown in these conditioned media. The process of vessel formation was observed for 24 to 48h after the addition of conditioned media. All the HMEC cells grown in glioma conditioned media showed extensive vessel formation after 48h. On the other hand, HMEC cells grown in co-culture media showed disorganized vessel formation or no vessel formation (Figure 1). To evaluate the mechanism of action of hUCBSC, we repeated the experiment with FAK inhibitor and siRNA to FAK (siFAK). In both the experiments, vessel formation by HMEC cells was highly disorganized compared to control glioma cells conditioned media. These results confirm that hUCBSC are acting by a mechanism similar to FAK inhibitor or siFAK. This proves our hypothesis that hUCBSC probably downregulate FAK in order to inhibit angiogenesis of glioma cells. To determine the effect of hUCBSC on the proteins involved in angiogenesis, we ran immunoblot analysis of lysates from single and co-cultures of glioma cells. FAK, pFAK, Akt and pAkt showed reduced expression in co-cultures (Figure 2A). Further, we tested the effect of hUCBSC on ERK1/2, pERK1/2. We observed that both these molecules were downregulated by hUCBSC treatments in co-cultures. We also tested the signaling molecule VEGF involved in angiogenesis and observed that stem cells decreased the expression of VEGF in glioma cells. These results prove that hUCBSC are able to downregulate proteins involved in angiogenesis, thereby preventing vessel formation. Similar to these results, transcriptional status of the molecules FAK, VEGF and VEGFR1 were also downregulated by hUCBSC (Figure 2B). In order to evaluate the specific effect of hUCBSC on glioma cells, we co-cultured glioma cells with human lung fibroblasts for 72h and ran immunoblot analyses for the above proteins. Fibroblasts did not show any effect on glioma cells in controlling their angiogenic proteins (Figure 2C). These results confirm that hUCBSC alone are effective in controlling the angiogenic proteins of glioma cells.

**Figure 3.**
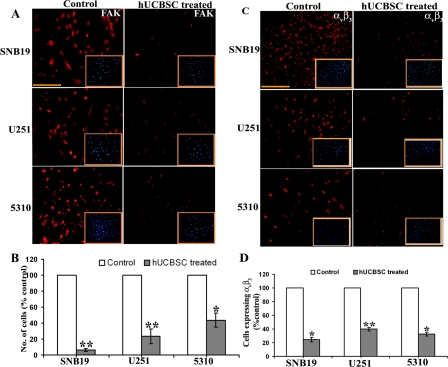
Detection of *in situ* expression of FAK and α_v_β_3_. (**A**) Single and co-cultures were maintained for 24h in 8-well chamber slides. 4×10^4^ of glioma cells in single cultures and 4×10^4^ glioma cells and 1×10^4^ of hUCBSC for co-cultures were seeded in each well. The cells were then processed for immunocytochemistry of FAK. Inset pictures show DAPI. (**B**) FACS analysis of cells expressing FAK. The single cultures and co-cultures were detached from the plates using Trypsin and gentle scraping followed by centrifugation at 1000rpm for 3min. The cells were then processed and analyzed for FAK expression using a flow cytometer (BD FACSCalibur). In each sample, 5000 cells were analyzed. (**C**) Detection of *in situ* expression of α_v_β_3_. The cells were processed for immunocytochemistry to check the expression of α_v_β_3._ Inset picture showing DAPI. (**D**) Quantitative estimation based on (C). Bar = 200μm. The results from three separate experiments are expressed as mean + SE. **p<0.01; *p<0.05.

FAK is a widely expressed cytoplasmic protein tyrosine kinase involved in integrin-mediated signal transduction and plays an important role in the control of several biological processes, including cell spreading, migration, survival and angiogenesis. We evaluated the expression of FAK by immunofluorescence and observed that hUCBSC inhibited the expression of FAK (Figure 3A). In co-cultures of glioma cells, the number of cells expressing FAK was very low as compared to control cells. Also, FACS analysis of single and co-cultures of glioma cells revealed that FAK expression was significantly inhibited by hUCBSC treatment (Figure 3B). This downregulation was significantly pronounced in co-cultures of SNB19 compared to U251 and 5310 co-cultures. Since FAK is associated with integrin signaling, we tested the expression of integrin α_v_β_3_ in glioma cells and their co-cultures. Similar to FAK expression, hUCBSC inhibited the expression of α_v_β_3_ in co-cultures (Figure 3C). Quantitative analysis revealed that SNB19 had a lower percentage of cells expressing α_v_β_3_ followed by 5310 and U251 (Figure 3D). To confirm these results, we isolated total RNA from single and co-cultures of glioma cells with hUCBSC and converted it into cDNA. We ran cDNA microarrays to test the array of genes related to angio-genesis that were affected by hUCBSC treatment. Most of the genes involved in angiogenesis were downregulated many times over by hUCBSC treatment (Table 1). In particular, Ang1 and Ang2 genes were downregulated apart from the growth factors EGF, FGF2, IGF1, PDGF-A and VEGF-A. This is associated with downregulation of Akt. These results confirm that hUCBSC influence a wide array of genes involved in angiogenesis, thereby controlling the growth of glioma cells.

**Table 1.** Effect of hUCBSC treatment on the expression of angiogenic molecules in glioma cells

Genes	Description	Fold difference		
		SNB19	U251	5310
AKT1	V-akt murine thymoma viral oncogene homolog 1	-1.32	-30.48	-1.24
ANG1	Angiopoietin 1	-3.36	-1.34	-2.28
ANG2	Angiopoietin 2	-1.33	-1.97	-6.02
EGF	Epidermal growth factor	-1.68	-24.76	-3.23
FGF2	Fibroblast growth factor 2	-1.19	-11.96	-1.56
IGF1	Insulin-like growth factor 1	-2.00	1.59	-3.12
MMP-2	Matrix metallopeptidase 2	-3.36	-27.47	-2.81
MMP-9	Matrix metallopeptidase 9	4389	10.7	317.37
PDGF-A	Platelet-derived growth factor alpha polypeptide	-1.32	-5.78	1.01
TGFB1	Transforming growth factor, beta 1	1.23	-19.43	1.47
THBS1	Thrombospondin 1	-1.37	-22.32	2.23
THBS2	Thrombospondin 2	-2.22	-5.39	-3.71
TIMP-1	TIMP metallopeptidase inhibitor 1	1.11	-15.78	-1.03
TIMP-2	TIMP metallopeptidase inhibitor 2	-1.15	-20.11	1.69
TIMP-3	TIMP metallopeptidase inhibitor 3	-2.31	4.99	2.85
VEGF-A	Vascular endothelial growth factor A	-4.59	-9.38	-3.34

Human Angiogenesis PCR arrays were run using cDNA from single and cocultures of glioma cells with hUCBSC. Real time PCR was carried out and changes in gene expression were illustrated as a fold increase/decrease according to manufacturer's instructions. The cut-off induction determining expression was 2.0 or -2.0 fold changes. Genes that met these criteria were considered to be upregulated or downregulated.

### Downregulation of FAK by hUCBSC treatment has an anti-angiogenic effect in U251 and 5310 glioma nude mice models

**Figure 4.**
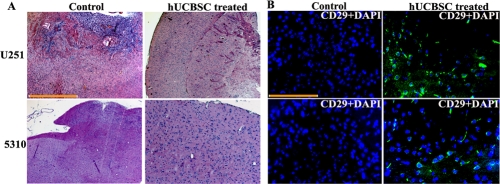
Intracranial regression of tumor growth by hUCBSC (**A**) Nude mice with pre-established intracranial human glioma tumors (U251 or 5310) were treated with hUCBSC by intracranial injection (2x10^5^). Fourteen days after hUCBSC administration, the brains were harvested, sectioned, and stained with Hematoxylin and Eosin. Bar = 2000μm (**B**) Characterization of hUCBSC in nude mice brain sections: Fourteen days after hUCBSC administration, the brains were harvested, sectioned and immunoprobed with mesenchymal stem cell markers CD29 using Alexa fluor-594 secondary antibody. (n = 3). Scale bar = 100μm.

**Figure 5.**
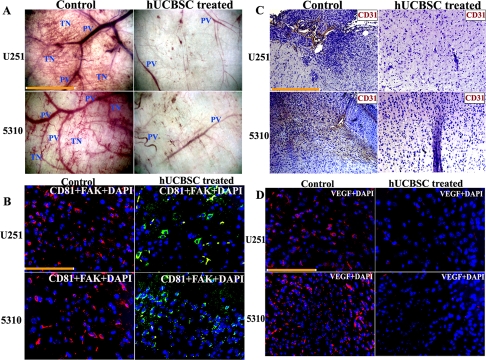
Inhibition of *in vivo* angiogenesis by hUCBSC. (**A**) Glioma cells, from single and co-cultures with hUCBSC, were suspended in serum-free medium and injected into the diffusion chambers. The chambers were sealed with bone wax and placed in serum-free medium to keep until used. Animals were anesthetized and 5mL of air was injected to create a dorsal skin sac. A 1.5-2 cm incision was made along the edge of the dorsal air sac. The chambers were then carefully placed through the incision under the skin, and the incision was closed. The animals were sacrificed after 2 weeks. The skin around the implanted chambers were carefully removed and observed under the phase contrast microscope. The novel (delicate, irregularly arising) branches were recorded as tumor-induced neovascularization. These branches were then compared to the more organized, pre-existing vasculature. PV=Pre-existing vasculature; TN=Tumor induced neovasculature. Bar = 50μm. (**B**) Immunofluorescence analysis for the downregulation of FAK. Orthotopic intracranial tumors were established in nude mice by injecting the glioma cells (U251 and 5310) and then treating with hUCBSC. The paraffin-embedded brain sections were subjected to immunofluorescence detection for (**B**) FAK and CD81 and (**D**) VEGF. FAK (anti-rabbit) is conjugated with goat anti-rabbit secondary antibody. CD81 (anti-goat) with FITC and VEGF (anti-mouse) was conjugated with Texas-red secondary antibodies. Bar=100μm. (**C**) DAB immunohistochemistry of CD31 expression in control and treated brain sections. Bar = 200μm.

Our *in vitro* experiments have proven that co-culture with hUCBSC can efficiently inhibit glioma angiogenesis by downregulating many genes involved in angiogenesis. Therefore, we investigated the anti-angiogenic effect of these stem cells *in vivo* using U251 and 5310 cells in nude mice. For this, we implanted U251 and 5310 cells into the right side of the brains of nude mice by intracranial administration as explained in Materials and Methods. After 7 days, hUCBSC were administered in a similar way but towards the left side of the brain. After the mice were implanted with U251, 5310 and hUCBSC, the mice were observed for 21 days. At that point, tumor samples were taken, and paraffin-embedded sections were prepared for immunohistopathological examination. Hematoxylin and Eosin (H&E) staining of the *in vivo* sections clearly showed that the tumors in hUCBSC-treated mice were inhibited significantly and were one-third the size of the tumors in control mice brains (Figure 4A). We checked for the presence of hUCBSC in the nude mice brains using mesenchymal stem cell markers CD29 by immunofluorescence. Control tumor sections did not show the expression of CD29. CD29^+^ cells were present only in the hUCBSC-treated brain sections, which confirm the presence of stem cells in the treated brains (Figure 4B). Further, we performed the dorsal skin fold chamber assay by introducing U251 and 5310 cells below the skin folds of athymic nude mice. In a similar fashion, co-cultures of both these cells with hUCBSC were also introduced below the skin folds of nude mice. The mice were euthanized after 14 days, and the skins were removed and observed under a bright field microscope. Extensive vessel formation was observed in the skins of mice treated with U251 and 5310 cells whereas skins having co-cultures of glioma cells with hUCBSC did not show vessel formation (Figure 5A). This *in vivo* assay proves that hUCBSC inhibited vessel formation under *in vivo* conditions.

We also checked the expression of FAK in the control tumor and hUCBSC-treated tumor sections. For this, we did co-localization experiments with CD81 (mesenchymal stem cell marker) and FAK antibody. FAK was highly expressed in the control tumor brains as compared to hUCBSC-treated brain sections, which showed high expression of CD81 (Figure 5B). We then checked the expression of CD31 or PECAM-1 (Platelet Endothelial Cell Adhesion Molecule-1) in both control tumors of hUCBSC-treated mice brain sections. DAB immunohistochemistry revealed that CD31 was highly expressed in both U251 and 5310 control tumor brains, whereas in hUCBSC-treated mice brains, expression of CD31 was almost negligible (Figure 5C). Next, we checked for expression of VEGF in the brain sections. Control tumor sections showed high expression of VEGF in the tumor areas, whereas in hUCBSC-treated tumors, very low level of expression of VEGF was observed (Figure 5D). These results confirm that FAK and VEGF expression were highly downregulated *in vivo* in hUCBSC-treated tumor brains.

**Figure 6.**
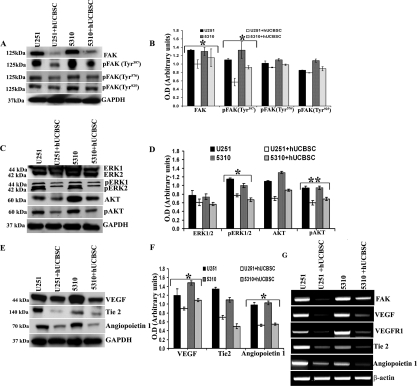
Inhibition of angiogenic molecules by hUCBSC *in vivo*. Immunoblot analysis for the expression of various angiogenic molecules in the tissue lysates of brains extracted from nude mice. Orthotopic intracranial tumors were established in nude mice by injecting glioma cells (U251 and 5310) and then treating with hUCBSC. Equal amounts of proteins (40μg) from untreated and treated mice brains were loaded on 8-14% gels and transferred onto nylon membranes, which were then probed with respective antibodies. GAPDH was used a positive loading control. (**A**, **C** & **E**) Western blots showing the downregulation of various angiogenic molecules by hUCBSC treatment. (**B**, **D** & **F**) Quantitative estimations of (**A**), (**C**) & (**E**). (G) Downregulation of various angiogenic molecules at the RNA level by hUCBSC. β-actin expression was used to serve as a loading control. The results from three separate experiments are expressed as mean + SE. **p<0.01; *p<0.05.

To evaluate the mechanism by which angiogenesis is inhibited in the nude mice tumor brains, we analyzed the tissue lysates by immunoblot analysis and observed that FAK was downregulated in tissue lysates of hUCBSC treatments. Then, we analyzed different phospho-forms of FAK, pFAK (Tyr^397^, Tyr^576^ and Tyr^925^). In hUCBSC-treated mice brains, downregulation of FAK and pFAK at Tyr^397^ was significant (Figure 6B). Out of all these phospho-forms of FAK, only pFAK (Tyr^397^) was significantly downregulated in hUCBSC treatments with no significant change in other phospho forms. This indicates that hUCBSC are targeting the phosphorylation of Tyr^397^ (Figure 6A). Other proteins that are involved in angiogenesis including ERK1/2, pERK1/2, Akt and pAkt were also downregulated in the hUCBSC treatments (Figure 6C). Quantification confirmed that these molecules were downregulated in tumor brains treated with hUCBSC (Figure 6D). Similar to our *in vitro* results VEGF, Tie2 and Ang1 were also downregulated under *in vivo* conditions (Figure 6E). Similar to immunohistochemistry results, VEGF was showing downregulation in tissue lysates also (Figure 6F). Finally, we evaluated the molecules that are involved in the process of angiogenesis by reverse-transcription based PCR. Growth factors VEGF, VEGFR1 along with FAK, Tie2 and Ang1 showed reduced expression, confirming that the process of inhibition by hUCBSC initiated at the transcription level (Figure 6G). Theseresults confirm that downregulation of FAK in mice tumor brains induces the downregulation of integrins as well as growth factors necessary for angiogenesis. Taken together, these results confirm the anti-angiogenic effect of hUCBSC *in vivo* and that tumor cell angiogenesis *in vitro* is efficiently inhibited by hUCBSC.

## DISCUSSION

Tumor angiogenesis, which is necessary for tumor cell invasion and proliferation, indicates an aggressive phase in tumor biology and typically correlates with increased tumor growth and metastasis [11]. Among all solid tumors, glioblastoma multiforme (GBM) has been reported to be the most angiogenic and displays the highest degree of vascular proliferation and endothelial cell hyperplasia [12]. Due to angiogenesis, the glioma vessel structure is markedly abnormal, resulting in decreased delivery of chemotherapies, increasing tumor hypoxia, and producing edema with its clinical consequences [13]. Targeting angiogenesis as a means of sensitizing gliomas to other treatment modalities has been adapted in recent times. Hence, we targeted angiogenesis of glioblastoma cells using hUCBSC. In our study, we observed that angiogenesis has been inhibited by hUCBSC in both *in vitro* as well as *in vivo* conditions. Our results provide hope that hUCBSC could become potential therapeutic tools to treat glio-blastoma.

Focal adhesion kinase (FAK) is an important mediator of growth factor signaling, cell proliferation, cell survival and cell migration. FAK resides at sites of integrin clustering—the so-called focal adhesions—that are prominent in cells grown in tissue culture [7]. FAK controls the dynamic regulation of integrin-linked adhesions, cadherin dependent cell-cell adhesions and peripheral actin structures, and so contributes to cell migration and invasion [5,14,15]. However, the causal role of FAK in tumor development combined with reports that increased FAK expression is associated with poor clinical outcome [8,9], indicate that FAK might be a useful therapeutic target [7,10].

FAK is overexpressed in invasive and metastatic tumors [16], and the FAK gene is also amplified in many types of tumors [17] suggesting a role for FAK in adhesion or survival in tumor cells. In cancer cells, attenuation of FAK expression induces detachment and apoptosis [18] suggesting that a FAK-dependent signal is required for tumor cell growth. We have previously shown that hUCBSC induces apoptosis in glioma cells [19] and upregulates PTEN in order to control their growth and proliferation [20]. In the present study, we also observed that downregulation of FAK resulted in decreased tumor growth in hUCBSC-treated tumors in mice brains. Here, we provide evidence suggesting that FAK promotes angiogenesis in malignant glioblastoma and hUCBSC downregulated FAK, thereby inhibiting vessel formation. We found an elevated expression of FAK in the glioma cells, which was highly downregulated by hUCBSC in the co-cultures. Furthermore, when these studies were repeated on brain tumors from an intracerebral nude mouse xenograft model of malignant glioblastoma, FAK and pFAK were detected at increased levels in the tumors as compared to the hUCBSC-treated brain tumors. These results suggest that hUCBSC regulate FAK levels (both normal as well as phosphorylated forms), and as such, hUCBSC could be used for the treatment of glioblastoma.

Integrins are transmembrane receptor molecules that are responsible for the interaction of endothelial and tumor cells with the ECM. Integrin signal transduction mediates endothelial cell migration and invasion [21]. The most common molecules allowing glioma cells to adhere to the ECM are integrins, particularly the integrin α_v_β_3_, which binds fibronectin in the ECM. In our study, we observed that α_v_β_3_is highly expressed in both *in vitro* as well as *in vivo* conditions (data not shown). Since hUCBSC were able to inhibit the expression of FAK, expression of α_v_β_3_ was also downregulated to significant levels both at transcription as well as translational levels. The downregulation of these molecules along with reduced expression of Akt and pAkt abolished the process of angiogenesis in tumors in the present study. Furthermore, the ability of tumor cells to migrate is demonstrated to be associated with increased FAK expression. The expression of FAK in human malig-nant astrocytic tumor cells increased their migration [22]. In contrast, inhibiting FAK expression and signaling reduced motility of adenocarcinoma cells [23]. The activation of endogenous EphA2 by ephrin-A1 was also found to suppress the activity of FAK and integrins [24], and thus inhibits integrin-mediated cell migration. Our results provide evidence that downregulation of FAK and phosphorylated FAK by hUCBSC resulted in decrease in the expression of VEGF which resulted in the attenuated migration of U251 and 5310 cells, and hence regression of tumor growth *in vivo.*

In normal brain tissue there is very little or no expression of VEGF and its receptor VEGFR-1. On the other hand, VEGF/VEGFR-1 is highly expressed in high-grade glioblastoma. Blocking or obstructing the VEGF/VEGFR signal transduction pathway by different methods can prevent the vascularization, metastasis, and edema of animal transplanted tumors and result in restricted tumor growth [25,26]. In the present study, there was expression of VEGF protein in glioma cells as well as each xenograft model of nude mice. hUCBSC could suppress the expression of VEGF and VEGF-A proteins in transplanted tumors in nude mice. We concluded that hUCBSC can lower the expression of receptor proteins VEGFR-1 and VEGFR-2 and weaken the signal transduction of VEGF. This can remarkably reduce the biological action of VEGF, thereby suppressing vessel formation.

We found that hUCBSC exhibit anti-angiogenic and anti-tumor activities both *in vitro* and *in vivo*. Our study shows that hUCBSC had the following key functions: [1] hUCBSC inhibited tube formation *in vitro*; [2] hUCBSC inhibited the development of new vessels in the dorsal skin fold chamber assay; and [3] hUCBSC inhibited the tumor growth induced by glioma cells. Moreover, hUCBSC inhibited the expression of FAK and related angiogenic molecules in the xenografts. In conclusion, our data suggest that FAK promotes angiogenesis in malignant glioblastoma tumors and that the mechanism by which FAK promotes angiogenesis involves integrin α_v_β_3_; hUCBSC were able to downregulate FAK and α_v_β_3_ as well as other signaling molecules involved in this process. Hence, hUCBSC could potentially be a useful therapeutic tool for the treatment of patients with glioblastoma.

## MATERIALS AND METHODS

### Ethics Statement

After obtaining informed consent, human umbilical cord blood was collected from healthy volunteers according to a protocol approved by the Peoria Institutional Review Board, Peoria, IL, USA. The consent was written and approved. The approved protocol number is 06-014, dated December 10, 2009. The Institutional Animal Care and Use Committee of the University Of Illinois College Of Medicine at Peoria, Peoria, IL, USA approved all surgical interventions and post-operative animal care. The consent was written and approved. The approved protocol number is 851, dated November 20, 2009.

### Cell Cultures

Two high-grade human glioma cell lines (SNB19 and U251) and a xenograft cell line (5310) were used for this study. SNB19 and U251 cells were obtained from ATCC. The xenograft cell line (5310) was kindly provided by Dr. David James at the University of California San Francisco. SNB19 and U251 cells were grown in Dulbecco's modified Eagle medium (DMEM) supplemented with 10% fetal bovine serum (FBS) and 1% penicillin-streptomycin. 5310 cells were grown in RPMI 1640 medium supplemented with 10% FBS and 1% penicillin-streptomycin. After obtaining informed consent, human umbilical cord blood was collected from healthy volunteers according to a protocol approved by the Institutional Review Board. Human umbilical cord blood was enriched by sequential Ficoll density gradient purification. Next, we selected cells using CD29^+^ and CD81^+^ markers as described previously [27]. The nucleated cells were suspended at a concentration of 1×10^6^/μL in Knockout DMEM (Invitrogen) supplemented with 10% FBS, 10% Knockout serum replacement (Invitrogen) and 1% penicillin-streptomycin and plated in 100-mm culture dishes. For co-culture experiments, hUCBSC and glioma cells were cultured at a ratio of 1:4. Co-cultures of hUCBSC and SNB19, hUCBSC and U251 were grown in DMEM; co-cultures of hUCBSC and 5310 were grown in RPMI-1640.Primary cultures of human lung fibroblasts were obtained from ATCC and were grown in DMEM supplemented with 10% FBS and 1% penicillin-streptomycin. Co-cultures of fibroblasts and SNB19, fibroblasts and U251 were grown in DMEM; co-cultures of fibroblasts and 5310 were grown in RPMI-1640.Cultures were maintained at 37°C in a humidified 5% CO_2_ atmosphere. In all experiments co-cultures were grown for 72h. Cultures were fed every 3 days with fresh medium.

### *In vitro* angiogenesis assay

The endothelial tube-like formation assay was performed as described previously [28]. Matrigel was plated into flat-bottomed 96-well tissue culture plates and then incubated at 37^o^C for 20min to allow the Matrigel to polymerize. Human microvascular endothelial cells (HMEC) were seeded in the matrigel-coated plates. After cells adhered to the wells (12h), growth medium was replaced by conditioned medium. Conditioned medium from SNB19, U251 and 5310 control and hUCBSC-treated glioma cells were added to HMEC cells. After 48h in the treated media, dead floating cells were washed away and the cells showing angiogenesis were stained by Hema-3 stain. We quantified tube formation by measuring the number of branch points and the total number of branches per point in triplicate wells.

### Dorsal skin fold chamber model

Tumor angiogenesis was quantified *in vivo* using the transparent dorsal skin fold chamber model [29]. Briefly, after total body anesthesia with ketamine (50mg/kg) and xylazine (5mg/kg), a dorsal air sac was made in the mouse by injecting 5 mL of air. After dorsal skin fold preparation, chambers were inoculated with 2×10^6^ glioma cells or co-cultures of glioma cells and hUCBSC.. 14 days later, the animals were anesthetized and sacrificed. The animals were carefully skinned around the implanted chambers, and the implanted chambers were removed from the subcutaneous air fascia. The skin fold covering the chambers was photographed under visible light. The number of blood vessels within the chamber in the area of the air sac fascia was counted, and their lengths were measured.

### Immunocytochemistry

Cultured hUCBSC were checked for mesenchymal markers by immuno-cytochemistry. Cultured cells plated in 2-well chamber slides were rinsed twice with phosphate buffered saline (PBS) and fixed in 4% paraformaldehyde. After additional PBS rinses, cells were blocked with 10% goat serum for 1h. Primary antibodies (1:100 dilutions) specific for mesenchymal markers [mouse anti-CD29 (Millipore), goat anti-CD81 (Santa Cruz)] were diluted in goat serum and applied overnight at 4^o^C. Texas Red conjugated anti-mouse or anti-rabbit secondary antibodies were diluted (1:200) in goat serum and applied individually for 1 to 2h at room temperature. Before mounting, the cells were stained with 4'. 6-diamidino-2-phenylindole (DAPI). In a similar manner single and co-cultures of glioma cells in 8-well chamber slides were processed with FAK and α_v_β_3_ antibodies. The cells were observed using a fluorescence microscope (Olympus IX71, Melville, NY) and/or a confocal microscope (Olympus Fluoview) and photographed.

### Construction of shRNA-expressing plasmid and transfection of siFAK

For this study, pSilencer™ 4.1-CMV plasmid vector (Ambion, Austin, TX) was used in the construction of the shRNA-expressing vector. The human FAK target sequenceAAGCCTTAACAATGC GTCAGTTTwas used for the siRNA sequence. Inverted repeat sequences were synthesized for FAK. The inverted repeats were laterally symmetrical making them self-complimentary with a 9-bp mismatch in the loop region. This 9-bp mismatch would aid in the loop formation of the shRNA. Oligonucleotides were heated in a boiling water bath in 6X SSC for 5 min and self-annealed by slow cooling to room temperature. The resulting annealed oligonucleotides were ligated to pSilencer at the BamHI and HindIII sites. For transfection, the cancer cells were cultured as mentioned previously. Cells at 60-70% confluency in 100mm tissue culture plates were transfected with 10μg of siRNA-expressing plasmid constructs (siFAK) using Fugene HD as per manufacturer's instructions (Roche).

### RNA extraction and quantitative real time PCR

All primer sequences were determined using established human GenBank sequences and designed using Primer3 software (v.0.4.0). For real time polymerase chain reaction (RT-PCR) analysis and RT-PCR-based micro-array analysis SA Biosciences, Frederick, MD), total RNA was isolated from control and hUCBSC-treated glioma cells and reverse transcribed into first strand cDNA. PCR amplification was performed using primer sets and amplified by 35 cycles (94^o^C, 1 min; 60^o^C, 1 min; 72^o^C, 1 min) of PCR using 20 pM of specific primers. Further quantitative analysis of genes was done by SYBR green based real-time PCR using Bio-Rad iCycler iQ Real-Time PCR Detection System. Each sample was measured in triplicate and normalized to the reference GAPDH or β-actin gene expression. Delta C_T_ (ΔC_T_) and DDC_T_ values were calculated and the fold change in the test gene expression was finally calculated. A statistical evaluation of real-time PCR results was performed using one-way analysis of variance (ANOVA) to compare test gene expression between cancer cells and their co-cultures with hUCBSC.

### Primers used for PCR

**Table:** 

FAK	Sense	5'ggtgcaatggagcgagtatt3'
	Antisense	5'gccagtgaacctcctctga3'
VEGF	Sense	5'ctacctccaccatgccaagt3'
	Antisense	5'cacacaggatggcttgaaga3'
VEGFR1	Sense	5'gttcaaggaacctcggacaa3'
	Antisense	5'gctcacactgctcatccaaa3'
Tie2	Sense	5'gttcacaagccttctcacacg3'
	Antisense	5'gttcacaagccttctcacacg3'
Ang1	Sense	5'acgatggcaactgtcgtgag3'
	Antisense	5'tccgacttcatgttttccacaa3'
β-Actin	Sense	5'gtcgtaccactggcattgt3'
	Antisense	5'cagctgtggtggtgaagct3'

### cDNA microarray analysis

We used human Angiogenesis PCR array (SA Biosciences) because of its advantage of real-time PCR performance combined with the ability of microarrays to detect the expression of many genes simultaneously. Real time PCR was carried out under the following conditions: one cycle of 95^o^C for 10 min, 40 cycles of 95^o^C for 15 sec and 60^o^C for 1 min. Changes in gene expression were illustrated as a fold increase/decrease according to manufacturer's instructions. The cut-off induction determining expression was 2.0 or -2.0 fold changes. Genes that met these criteria were considered to be upregulated or downregulated. We performed these experiments in duplicate.

### Immunoblot analysis

Single and co-cultures of glioma cells or nude mice brain tissues were harvested and homogenized in four volumes of homogenization buffer and processed for cell lysates as described previously [27]. Samples (40μg of total protein) were subjected to 8-14% SDS-PAGE and transferred onto nitrocellulose membranes. The reaction was detected using Hyperfilm-MP autoradiography film (Amersham, Piscataway, NJ). The following antibodies were used for Western blot analysis: mouse anti-FAK (1:200; Santa Cruz Biotechnology, Santa Cruz, CA), mouse anti-pFAK and all other pFAK antibodies (1:500; Santa Cruz), goat integrin α_v_β_3_ (1:500; Santa Cruz), mouse anti-ERK (1:5000; BD Biosciences, Franklin Lakes, NJ), rabbit anti-AKT (1:1000; Cell Signaling), mouse anti-Ang1 (1:1000; Cell Signaling), rabbit anti-VEGF [Phospho-Rac1/cdc42 (Ser^71^) antibody (1:1000; Cell Signaling], and mouse anti-Tie2 (1:1000; BD Biosciences). Immunoblots were stripped and redeve-loped with GAPDH antibody [mouse anti-GAPDH (1:1000; Santa Cruz)]to ensure equal loading levels. Experiments were performed in triplicate. Values for treated and untreated samples were compared using one-way ANOVA. A *p* value of <0.05 was considered significant.

### Intracranial tumor growth

The Institutional Animal Care and Use Committee of the University Of Illinois College Of Medicine at Peoria approved all surgical interventions and post-operative animal care. U251 (1×10^6^ cells) and 5310 (8×10^5^ cells) tumor cells were intracerebrally injected into the right side of the brains of nude mice, as described previously [30]. Seven days after tumor implantation, the mice were injected with hUCBSC near the left side of the brain. The ratio of the hUCBSC to glioma cells was maintained at 1:4. Three weeks after tumor inoculation, six mice from each group were sacrificed by cardiac perfusion with 4% formaldehyde in PBS, their brains were removed, and paraffin sections were prepared. Sections were stained with H&E to visualize tumor cells and to examine tumor volume. The sections were blind reviewed by a neuropathologist and scored semiquantitatively for tumor size. Whole-mount images of brains were also taken to determine infiltrative tumor morphology. The average tumor area per section integrated to the number of sections where the tumor was visible was used to calculate tumor volume; tumor volumes were compared between controls and treated groups.

### Immunohistochemical analysis

Brains of control and hUCBSC-treated mice brains were fixed in formaldehyde and embedded in paraffin as per standard protocols. Sections were deparaffinized as per standard protocol. After H&E staining, transmitted light images were obtained to visualize the morphology of the sections. For immunohistochemistry, sections were blocked in 10% goat serum for 1h, and were subsequently transferred to primary antibody diluted in 10% goat serum (1:100) overnight at 4^o^C in a humidified chamber. Sections were then washed in 1% BSA in PBS, incubated with the appropriate secondary antibody for 1h and visualized using a fluorescence microscope. For immunofluorescence, sections were treated with primary antibodies overnight at 4^o^C and then treated with appropriate Alexa fluor secondary antibodies at room temperature for 1h. Negative controls were maintained either without primary antibody or using IgG.

### Statistical analyses

Quantitative data from cell counts, Western blot analysis, and other assays were evaluated for statistical significance using one-way analysis of variance (ANOVA). Data for each treatment group were represented as mean ± SE and compared with other groups for significance by one-way ANOVA followed by Bonferroni's post hoc test (multiple comparison tests) using Graph Pad Prism version 3.02, a statistical software package. Results were considered statistically significant at a p value less than 0.05.

## Abbreviations

ERK: Extracellular signal-regulated kinases
EGF: Epidermal growth factor
FAK: focal adhesion kinase
FACS: Fluorescence-Activated Cell Sorting
FGF2: Basic fibroblast growth factor
hUCBSC: human umbilical cord blood stem cells
HMEC: Human microvascular endothelial cells
IGF: Insulin-like growth factor
PDGF: Platelet-derived growth factor
VEGF: Vascular endothelial growth factor
